# Editorial: Gut microbiota as a weapon against infections

**DOI:** 10.3389/fcimb.2023.1277517

**Published:** 2023-08-24

**Authors:** Shikha Negi, Susanta Pahari

**Affiliations:** ^1^ Department of Immunobiology, Cincinnati Children’s Hospital Medical Centre, Cincinnati, OH, United States; ^2^ Host Pathogen Interactions Programs, Texas Biomedical Research Institute, San Antonio, TX, United States

**Keywords:** microbiota, fecal microbiota transplantation, infection, gut-lung axis, probiotics, tuberculosis

Gut microbiota plays a dominant role in host defense against infections. The Research Topic entitled “*Gut microbiota as a weapon against infections*” in the *Intestinal Microbiome* section is centered on new perspectives, recent advances, and current challenges in microbiome and infection. This Research Topic is focused on the significant role and potential of gut microbiota in combating infections and improving overall human health. Seven original research articles and two reviews were published on this Research Topic.

Microbiota colonization during early life is crucial for infant health and immunity. Huang et al. aimed to understand the mechanisms of antibiotic-induced fungal infection in preterm infants. They found that broad-spectrum antibiotics may promote invasive fungi disease (IFD) in preterm rats. A decreased abundance of beneficial gut microbes such as Clostridium species and *Bacteroides* accompanies this IFD. This study highlights the importance of symbiotic microbiota, which may reduce the risk of IFD. Further, Cong et al. reviewed the potential of intestinal bacteria to combat invasive fungal infections. Here, they discussed related research studies and emphasis on the ability of intestinal commensal bacteria and probiotics to restrict the invasion of pathogenic fungi.

The 16S rRNA microbial profiling approach was employed by Lee et al. to determine the microbiota composition in *Clostridioides difficile* infections (CDI) patients after high-dose vitamin D supplementation. Interestingly, they found an elevated abundance of beneficial intestinal bacteria such as *Bifidobacteriaceae* and *Christensenellaceae*. However, a large cohort study is needed to establish the potential of vitamin D as replacement therapy in patients with CDI.

In the investigation by Zhang et al. the potential role of lactic acid bacteria (LAB) in preventing yak disease-related diarrhea was demonstrated. As an essential animal species on the plateau, Yak is an important source of livelihood and economy for herders. However, yak diarrhea, especially calf diarrhea, has brought substantial economic losses to residents. In this study, authors examine the probiotic potential of four species of lactic acid bacteria to prevent/treat yak diarrhea. The findings from this article can be accommodative to the meta-analysis work in the future and may formulate good probiotic clues. Chen et al. investigated how Cyprinid herpesvirus 2 (CyHV-2) infection impacts the gut microbiota of gibel carp. Their metabolome approach found a decrease in microbiota-regulated metabolites upon infection which correlated with the changes in the bacterial community.

Fecal microbiota transplantation (FMT) is an effective treatment for gastrointestinal disorders and highlights the importance of healthy microbiota in treating diseases. Zou et al., research on pediatric Crohn’s disease (CD) patients reported that FMT in conjugation with partial enteral nutrition could be used as a first-line treatment for active CD in children. They did not consider the gut flora variation in these patients, which warrants future prospective studies.

Better knowledge of the interplay between microbiota and drugs can help develop effective disease therapies to treat diseases. The study by Wan et al. evaluated the effect of the first-class hypoglycemic drug metformin on sepsis-related liver lung in aged rats. They highlighted in their study that metformin could alleviate inflammatory response and lung injury by reversing the imbalanced gut microbiota composition. These results provide a potential treatment for sepsis-associated acute lung injury (SALI) in aged rats with sepsis.

Another critical aspect to consider is the gut-lung axis, which indicates the crosstalk between the gut and lung that can impact infections such as tuberculosis (TB). Ye et al. cross-sectional study revealed that pulmonary tuberculosis patients exhibit significantly different microbiota from healthy controls. This finding suggests that altered gut microbiota might be the possible fundamental pathophysiology of pulmonary tuberculosis. Another interesting review article by Yu et al. summarized the current findings regarding tuberculosis and the gut microbiome. They have discussed studies related to the alteration of the gut microbiome in patients with pulmonary TB (PTB) and intestinal TB (ITB). They have focused on establishing *Mycobacterium tuberculosis* in the gastrointestinal tract and potential probiotics as well as postbiotics in treating ITB in PTB patients. These studies add to the knowledge of the gut-lung axis that can be a therapeutic target to improve TB infection.

In conclusion, our Research Topic provides some appreciable insights into the crucial role of gut microbiota in controlling bacterial and fungal infections. Thus, the published articles on this Research Topic will bring the opportunities to understand the potential role of commensal microbiota and their mechanisms to modulate host immunity, which could contribute to novel microbiota based therapeutics to treat infections.

## Author contributions

SN: Conceptualization, Writing – original draft, Writing – review & editing. SP: Writing – original draft, Writing – review & editing.

